# Enterovirus 71 Virion-Associated Galectin-1 Facilitates Viral Replication and Stability

**DOI:** 10.1371/journal.pone.0116278

**Published:** 2015-02-23

**Authors:** Pei-Huan Lee, Chia-Ming Liu, Tzong-Shiann Ho, Yi-Che Tsai, Chi-Cheng Lin, Ya-Fang Wang, Yuh-Ling Chen, Chun-Keung Yu, Shih-Min Wang, Ching-Chuan Liu, Ai-Li Shiau, Huan-Yao Lei, Chih-Peng Chang

**Affiliations:** 1 Department of Microbiology & Immunology, College of Medicine, National Cheng Kung University, Tainan, 701, Taiwan; 2 Department of Emergency Medicine, National Cheng Kung University Hospital, Tainan, 701, Taiwan; 3 Department of Oral Medicine, College of Medicine, National Cheng Kung University, Tainan, 701, Taiwan; 4 Department of Pediatrics, National Cheng Kung University Hospital, Tainan, 701, Taiwan; 5 Center of Infectious Disease and Signaling Research, National Cheng Kung University, Tainan, 701, Taiwan; 6 National Institute of Infectious Diseases and Vaccinology, National Health Research Institutes, Zhunan, 350, Taiwan; University of North Carolina School of Medicine, UNITED STATES

## Abstract

Enterovirus 71 (EV71) infection causes a myriad of diseases from mild hand-foot-and-mouth disease or herpangina to fatal brain stem encephalitis complicated with pulmonary edema. Several severe EV71 endemics have occurred in Asia-Pacific region, including Taiwan, and have become a serious threat to children’s health. EV71 infection is initiated by the attachment of the virion to the target cell surface. Although this process relies primarily upon interaction between viruses and cell surface receptors, soluble factors may also influence the binding of EV71 to host cells.Galectin-1 has been reported to participate in several virus infections, but is not addressed in EV71. In this study, we found that the serum levels of galectin-1 in EV71-infected children were higher than those in non-infected people. In EV71 infected cells, galectin-1 was found to be associated with the EV71 VP1 and VP3 via carbohydrate residues and subsequently released and bound to another cell surface along with the virus. EV71 propagated from galectin-1 knockdown SK-N-SH cells exhibited lower infectivity in cultured cells and less pathogenicity in mice than the virus propagated from parental cells. In addition, this galectin-1-free EV71 virus was sensitive to high temperature and lost its viability after long-term storage, which could be restored following supplement of recombinant galectin-1. Taken together, our findings uncover a new role of galectin-1 in facilitating EV71 virus infection.

## Introduction

Enterovirus 71 (EV71) is a human enterovirus in the *Enterovirus* genus of the *Picornaviridae* family. EV71 infection was first recognized in 1974 in the United States[1]. Subsequent outbreaks were reported in Australia, Sweden, Japan, Bulgaria, Hungary, Hong Kong, and Malaysia. Several severe EV71 endemics have since occurred in Taiwan and the Asia-Pacific region, and EV71 has emerged as a serious threat to public health in the Western Pacific region[2–5]. EV71 infection primarily causes hand-foot-mouth disease (HFMD) in young children. Most patients are febrile at diagnosis and some even present with a temperature exceeding 39 degrees. Live EV71 virus can be easily cultured from these febrile patients, indicating that EV71 may be resistant to high temperature[3]. In addition to HFMD and herpangina, severe neurological complications such as meningitis, poliomyelitis-like syndrome, and fatal pulmonary edema have also occurred occasionally. Brain stem encephalitis is the cardinal feature of EV71 central nervous system (CNS) involvement and is also associated with high mortality (about 26%)[3]. The pathogenesis of EV71 infection is not clear and there is, as yet, no available vaccine or effective antiviral agent.

EV71 is a non-enveloped virus with icosahedral structure built by capsid proteins known as VP1, VP2, VP3 and internal VP4. In addition to 4 structural proteins, there are another 7 non-structural proteins (2A, 2B, 2C, 3A, 3B, 3C and 3D) which are responsible for EV71 virus replication as encoded in the EV71 genome[6]. In order to successfully replicate in host cells, viruses usually require host factors to facilitate their replication. Emerging reports have found several host proteins participating in EV71 replication. Heterogeneous nuclear ribonucleoprotein A1 (hnRNP A1), and far-upstream element-binding proteins 1 and 2 (FBP1 and FBP2) have been shown to regulate EV71 internal ribosome entry site (IRES) activity[7–9]. Both hnRNP K and reticulon 3 can enhance viral RNA synthesis[10,11]. Heat shock protein-90 beta (HSP 90β) has recently been reported to support EV71 viral particle assembly and is associated with released virions instead of promoting viral replication[12]. On the other hand, EV71 can interfere with host mRNA polyadenylation by cleaving the cleavage stimulation factor 64 subunit (CstF-64) that is in turn beneficial for its own replication[13]. These studies not only point to the importance of host factors in EV71 replication but also provide potential antiviral targets.

Galectin-1 has been identified as a soluble beta-galactoside binding lectins and its functions are dependent on multivalent glycan binding[14]. The expression of galectin-1 is abundant in brain and lymphoid tissues, and it has been shown to regulate multiple neuron degeneration and immune responses[15]. Recent reports have revealed that galectin-1 can be upregulated by bacteria or virus infection. The free form galectin-1 can interact with these viruses either enhancing their binding to host cells, or interfering their maturation [16–19]. The role of galectin-1 in EV71 infection and pathogenesis is unclear. In this study, we report that the serum level of galectin-1 in patients is increased. Galectin-1 is found to bind EV71 during viral replication and also on released virions in a cultured neuron cell line. The virion-associated galectin-1 is crucial for EV71 to infect new target cells and animals hosts, and to facilitate virus against thermal and environmental stress. We draw attention to how that galectin-1 contributes to EV71 replication and disease pathogenesis.

## Results

### EV71 binds to galectin-1

**Fig 1:**
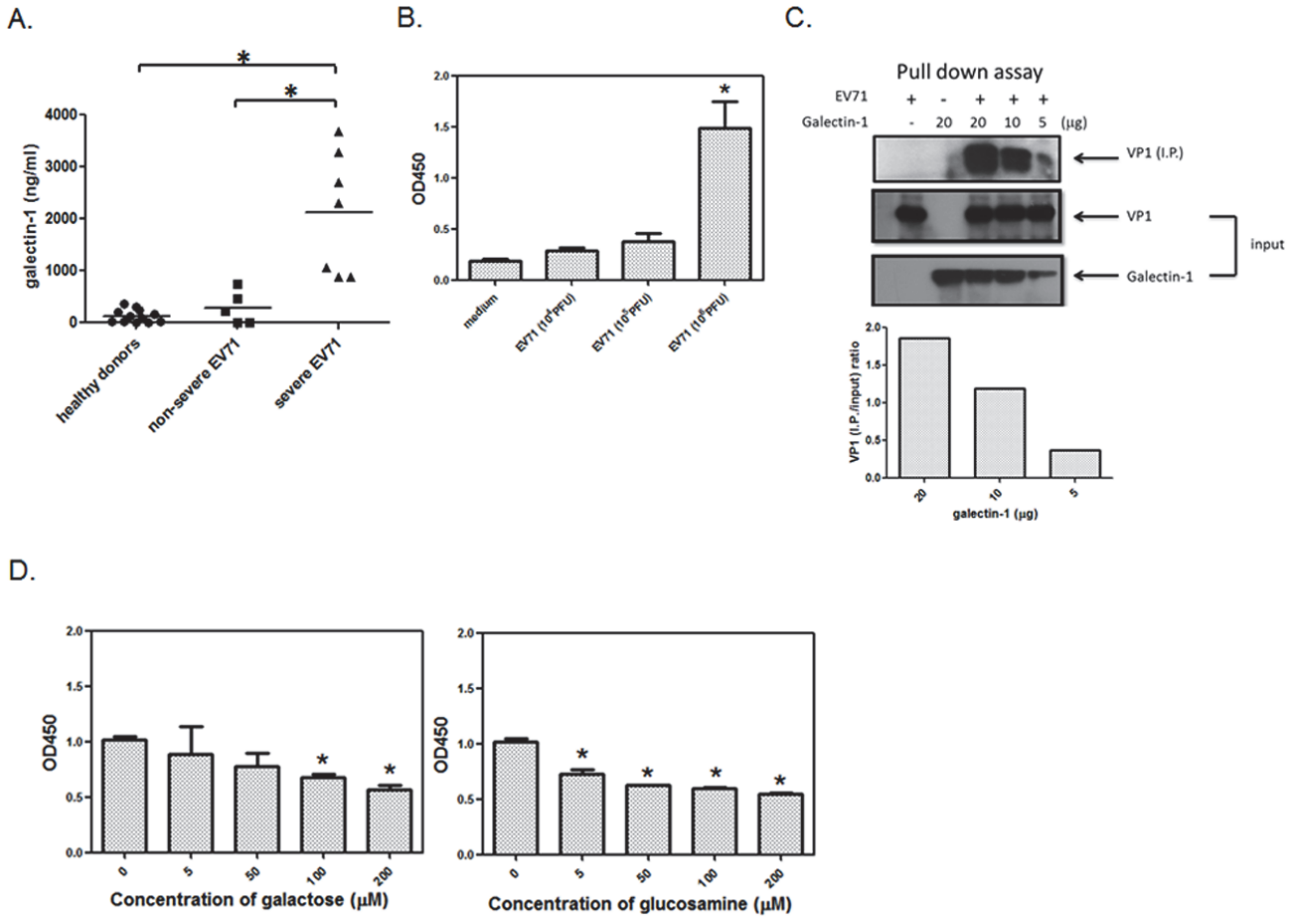
EV71 viruses bind to galectin-1. (A) Galectin-1 serum levels are increased in EV71-infected patients. Seven clinical severe EV71-infected patient sera, five non-severe EV71-infected patient sera and twelve non-infected control sera were collected to detect galectin-1 by ELISA. *p<0.05 by Kruskal-Wallis test. (B) EV71 viruses are captured by galectin-1. EV71 viruses in 10 fold serial dilution from 10^6^ PFU to 10^4^ PFU were added to galectin-1-coated ELISA plate. The bound viruses were detected by anti-EV71 antibody and HRP-conjugated goat anti-mouse IgG antibody. *p<0.05 compared to medium group by one-way ANOVA followed by Tukey correction. (C) EV71 viruses are pulled down with galectin-1. EV71 viruses (1×10^6^ PFU) were incubated with 5, 10, 20 μg recombinant galectin-1 at 4°C overnight. The EV71/galectin-1 complexes were then pulled down by using TALON metal affinity resins. The protein level of pulled down VP1 of EV71was detected by Western blotting and further normalized with input VP1 of EV71. This representative result was from one of two independent experiments. (D) Galactose and N-acetyl-glucosamine inhibit EV71 viruses binding to galectin-1. EV71 viruses were pre-incubated with galactose or N-acetyl-glucosamine at 4°C for 2 h and then added to galectin-1-coated ELISA plate1 at 37°C for another 2 h. The bound viruses were detected by anti-EV71 antibody and HRP-conjugated goat anti- mouse IgG antibody. *p<0.05 compared to non-adding carbohydrates group by one-way ANOVA followed by Tukey correction.

Lectin is a specific carbohydrate binding protein. A recent study demonstrated that cell surface glycosylation may affect EV71 binding to host cells[20], indicating that carbohydrate binding proteins as lectins may participate in the interaction between EV71 virus and host cells. Thus, we first examined whether EV71 can bind to plant lectins through different carbohydrate binding moieties. Lectins such as Concanavalin A (Con A, mannose or glucose-specific), Lens culinaris agglutinin (LCA, mannose or glucose-specific), Ricinus communis agglutinin (RCA, N-acetyl galactosamine or galactose-specific), Wheat germ agglutinin (WGA, N-acetyl glucosamine or sialic acid-specific), or Dolichos biflorus agglutinin (DBA, N-acetyl galactosamine-specific) were coated on the plate to capture the EV71 virions. The bound EV71 was detected by anti-EV71 mAb and second anti-mIgG-HRP conjugate. The Con A, LCA, RCA, WGA, and DBA lectins can all capture the EV71 (S1 Fig.). It seemed that EV71 might contain various types of carbohydrates on the virions to bind lectins. Galectin-1 is a galactose-specific binding lectin in mammalian cells. Moreover, we found that an extreme upsurge in serum galectin-1 was seen in severe EV71-infected children (Fig. 1A). Hence we further investigated the interaction efficiency of galectin-1 with EV71 virus. Using a recombinant galectin-1coated ELISA plate, the EV71 virions were found to bind galectin-1 at virus titer up to 10^6^ PFU (Fig. 1B). To further confirm the binding in solution between galectin-1 and EV71, the EV71 stock was mixed with recombinant his-tagged galectin-1 in solution, which was eventually separated by TALON metal affinity resins, and the pull-down EV71 was detected by anti-VP1 antibody on Western blot. As shown in Fig. 1C, galectin-1 can pull down the EV71 in a dose-dependent manner. This binding has carbohydrate specificity, as galactose and N-acetyl-glucosamine can competitively inhibit the EV71 binding to galectin-1 (Fig. 1D). These data confirmed that EV71 can bind to several different lectins, including galectin-1.

### Galectin-1 interacts with EV71 virus in infected host cells and also on released virions

**Fig 2:**
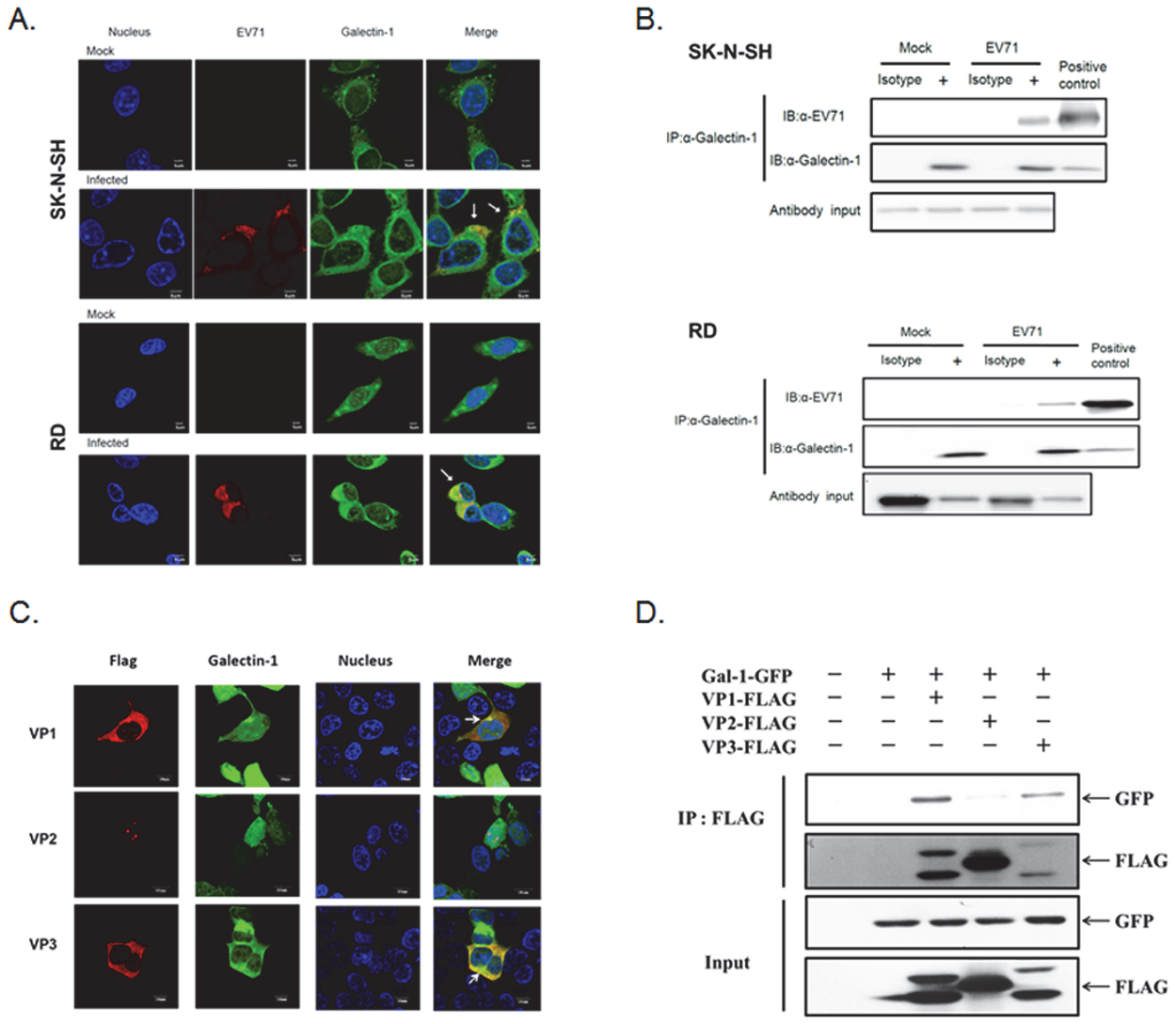
EV71 viruses interact with cellular galectin-1 in EV71-infected cells. (A) Co-localization of galectin-1 and EV71 in EV71-infected cells. SK-N-SH or RD cells were infected with EV71 viruses with MOI = 1 for 8 h and then stained with Hoechst 33258, anti-EV71, and anti-galectin-1 antibodies. The distributions of these proteins were analyzed by confocal microscopy. The co-localization of EV71 and galectin-1 is indicated by arrows. (B) Co-immunoprecipitation of galectin-1 and EV71virus from EV71-infected cells. SK-N-SH and RD cells were infected with EV71 virus with MOI = 1 for 8 h. Cells were lysed and then precipitated by anti-galectin-1 antibody. The precipitated proteins were analyzed by Western blotting. EV71 viruses input as positive control. (C) Galectin-1 interacts with VP1 and VP3 of EV71. 293T cells were co-transfected with pEGFP-galectin-1 and pFLAG-MSCV (VP1, VP2 or VP3) for 24 hours. The cells were fixed and stained with anti-Flag antibody. The colocalization of three VP proteins and galectin-1 was detected by confocal microscopy. The arrows indicate colocalization of the VP protein and galectin-1. (D) Co-immunoprecipitation of galectin-1 and EV71 VP proteins. Galecin-1-GFP, VP1-FLAG, VP2-FLAG and VP3-FLAG plasmids were introduced to 293T cells. Cells were lysed and then precipitated by anti-FLAG antibody. The precipitated proteins were detected by Western blotting. Results are representative of two independent experiments.

**Fig 3:**
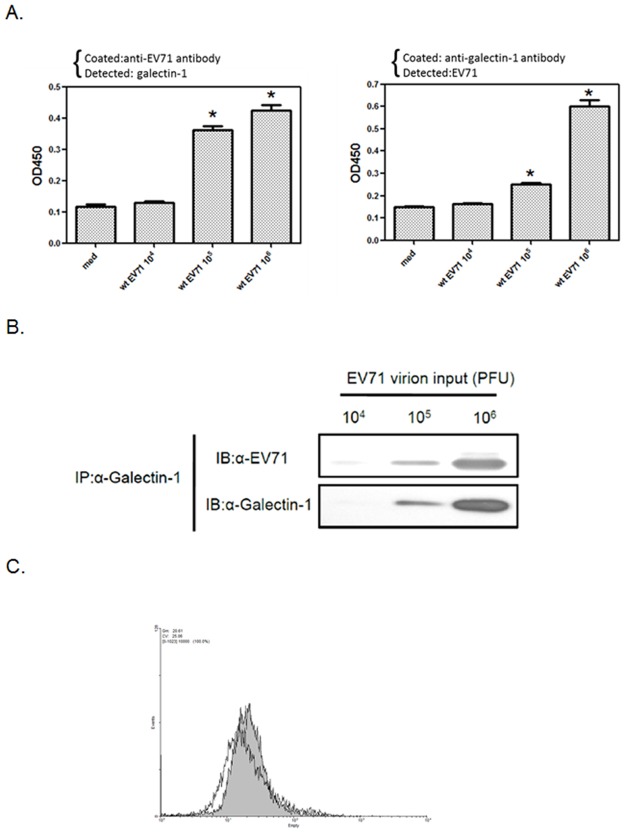
Galectin-1 is associated with released EV71 virions. (A) Detection of galectin-1/EV71 virion complexes by ELISA. EV71 viruses in 10-fold serial dilutions from 10^6^ to 10^4^ PFU were added to anti-EV71 antibody-coated (left panel) or anti-galectin-1 antibody-coated (right panel) 96-well plates to detect galectin-1 or EV71, respectively. *p<0.05 compared to medium group by one-way ANOVA followed by Tukey correction. (B) Immunoprecipitation of galectin-1 with EV71 virions. EV71 virions (10^4^, 10^5^, 10^6^ PFU) were incubated with anti-galectin-1 antibody at 4°C overnight. After purification by protein G-agarose beads, the precipitated proteins were analyzed by Western blotting. (C) Galectin-1 to bind SK-N-SH cells alone with EV71 viruses. SK-N-SH cells were incubated with medium (mock) or EV71 viruses (MOI = 100) at 4°C for 1 hour. The surface galectin-1 of mock or EV71-incubated SK-N-SH cells was determined by flow cytometry. Results are representative of two (C) or three (A, B) independent experiments.

We next examined whether the binding between galectin-1 and EV71 virus had occurred in EV71-infected cells. Human neuroblastoma (SK-N-SH) or rhabdomyosarcoma (RD) cells were infected with EV71 virus to monitor the interaction of virus and galectin-1. Using immunofluoresce staining, galectin-1 was found to colocalize with EV71 virus in SK-N-SH or RD cells under a conforcal microscope (Fig. 2A). To further confirm the interaction between galectin-1 and EV71, the co-immunoprecipitation of galectin-1 and EV71 was performed on EV71-infected cells. As shown in Fig. 2B, the anti-galectin-1 antibody can precipitate the EV71-galectin-1 complexes that were detected with immunoblot by anti-EV71 as well as anti-galectin-1 antibody. These are found for both SK-N-SH and RD cells, indicating that galectin-1 interacts with EV71 virus during replication. To further determine which EV71 capsid protein is the binding target of galectin-1, each FLAG-VP1,-VP2 or-VP3 protein construct was co-transfected with GFP-galectin l construct to 293T cells. The protein-protein interactions were determined by confocal microscopy. Both VP1 and VP3, but not VP2, showed both co-localization and co-immunoprecipitation with galectin-1 in 293 T cells (Fig. 2C and D), suggesting that galectin-1 binds EV71 virus via VP1 and VP3. Since EV71 virus can bind galectin-1, we further determined whether galectin-1 is associated with released EV71 virions. The released EV71 virions were collected from supernatants of EV71- infected SK-N-SH cells and then captured and detected by anti-galectin-1 or anti-EV71 antibody-based ELISA. It was found that galcetin-1/EV71 virion complexes were captured by either anti-galectin-1 or anti-EV71 antibody (Fig. 3A). These galectin-1 containing EV71 virions were also precipitated by anti-galectin-1 antibody (Fig. 3B). To further determine whether the galcetin-1/EV71 virion complexes can bind to host cells, the collected EV71 virions were incubated with SK-N-SH cells at 4°C to monitor the cell-surface-bound galectin-1.As shown in Fig. 3C, the surface-bound galectin-1 on SK-N-SH cells was increased after incubating with EV71, indicating that galectin-1 is coupled with these EV71 virions. These results demonstrate that cellular galectin-1 can interact with EV71 virus not only during replication within host cells but also on released virions.

### Virion-associated galectin-1 contributes to EV71 viral infectivity and virulence

**Fig 4:**
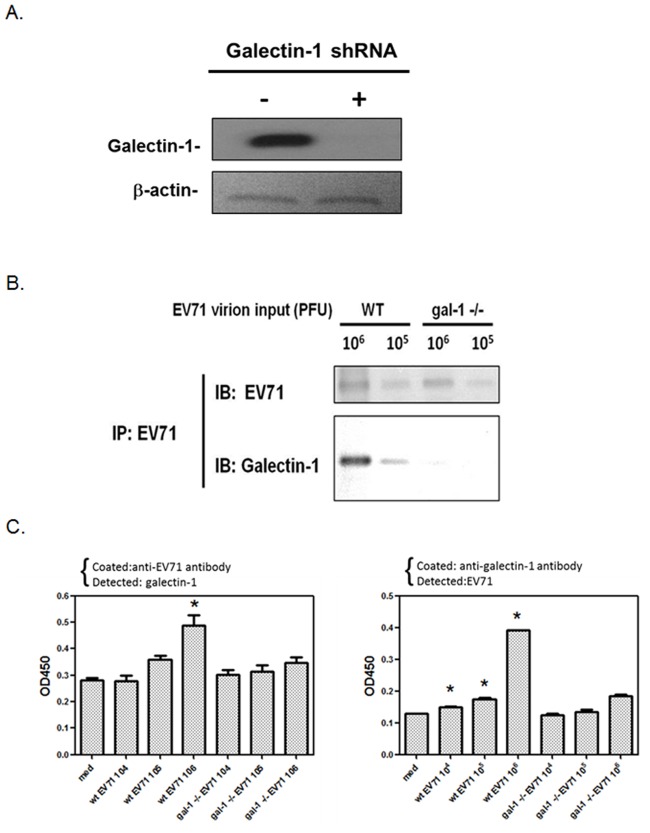
EV71 viruses propagated from galectin-1 silenced cells do not carry galectin-1. (A) Galectin-1 is silenced by lentiviral vector-based shRNA. SK-N-SH cells were infected with lentiviruses encoding a galectin-1 shRNA for 48 h. The silencing of galectin-1 in SK-N-SH cells was determined by Western blotting. (B) Co-immunoprecipitation of WT EV71 or galetin-1 -/- EV71 virus with galectin-1. WT or galectin-1 -/- EV71 viruses were immunoprecipitated by anti-EV71 antibody. The expressions of galectin-1 and EV71 were analyzed by Western blotting. (C) Detection of galectin-1 on WT or galectinl-1 -/- EV71 viruses by ELISA. EV71 in 10 fold serial dilutions from 10^6^ to 10^4^ PFU was added to anti-EV71 antibody-coated (left panel) or anti-galectin-1 antibody-coated (right panel) 96-well plate to detect galectin-1 or EV71, respectively. *p<0.05 compared to medium group by one-way ANOVA followed by Tukey correction. Results are representative of three independent experiments.

**Fig 5:**
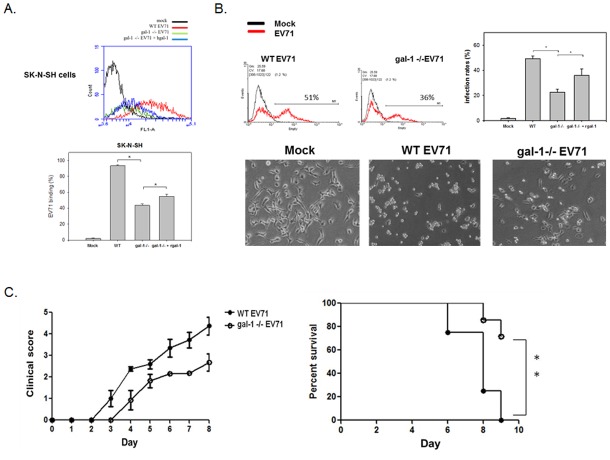
Galectin-1-/- EV71 shows less cellular binding, infectivity, mice neurological syndromes, and mortality. (A) Galectin-1-/- EV71 viruses reduce their cell binding activity. SK-N-SH cells were incubated with WT, galectin-1 -/- EV71 or galectin-1 -/- EV71 viruses with recombinant galectin-1 (25 ng/ml) at 4°C (MOI = 100) for 3 h. The surface-bound EV71 was detected by anti-EV71 antibody and hence analyzed by flow cytometry. *p<0.05 by one-way ANOVA followed by Tukey correction.(B) Galectin-1-/- EV71 viruses show lower infectivity than WT viruses. SK-N-SH cells were infected with WT, galectin-1 -/- EV71 or galectin-1 -/- EV71 viruses with recombinant galectin-1 (25 ng/ml) at 37°C (MOI = 0.5) for 12 h. The EV71-infected cells were analyzed by anti-EV71 antibody via flow cytometry. The virus-induced cytopathic effect was monitored under a microscope. *p<0.05 by one-way ANOVA followed by Tukey correction.(C) Galectin-1-/- EV71 viruses cause less neurological syndromes and mortality in mice. One week-old C56BL/6 mice were infected with 10^6^ PFU of WT (n = 7) or galectin-1 -/- EV71 (n = 6) by intraperitoneal injection. The clinical scores and survival rate were monitored daily post-infection. ** p<0.01 by the log-rank (Mantel-Cox) test. Results are representative of two (A, B) or three (C) independent experiments.

In order to determine the role of virus-associated glaectin-1 on EV71 infection, we tried to generate galectin-1-/- EV71 virus from galectin-1-deficinet cells. Using a lentiviral vector containing galectin-1 shRNA, the galectin-1 was successfully silenced in SK-N-SH cells (Fig. 4A). The EV71 viruses propagated from galectin-1 silenced SK-N-SH cells showed similar viral structure as wild type viruses by electronic microscopy observation (S2 Fig.), and were then collected to detect virus-associated galectin-1. Compared to those from wild type cells, EV71 viruses propagated from glaectin-1 silenced cells did not contain any galectin-1 protein, as confirmed by both antibody-based precipitation assay and ELISA (Fig. 4B and C). Next, we proceeded by examining the infectivity and virulence of the galectin-1-free EV71 viruses. We found that galecetin-1-/- EV 71 viruses showed a lower binding ability to host cells compared with the wild type virus (Fig. 5A). They also infected fewer SK-N-SH cells and caused a milder cytopathic effect (Fig. 5B), indicating that the galectin-/- EV71 viruses presented reduced infectivity. Such a marked decline in cell binding and infectivity of galectin-1-/- EV71 viruses was recovered by a supplement of recombinant galectin-1 (Fig. 5A and B).To evaluate the virulence of the virus, one week-old C56BL/6 mice were infected with either wild type or galectin-1-/- EV71 viruses to monitor virus-caused mice neuropathological symptoms and mortality. The mice infected with wild type EV71 viruses showed initial clinical signs of hunchback and wasting from day 3 after infection and further suffered from limb weakness and paralysis with the progressing number of days, and almost all died by around day 9. However, those mice which were infected with galectin-1-/- EV71 viruses presented mild neuropathological symptoms and a high survival rate (70%) (Fig. 5C). These results indicate that EV71 virus-coupled galectin-1 can promote viral infectivity and virulence.

### Galectin-1 facilitates EV71 viruses against thermal and environmental stress

**Fig 6:**
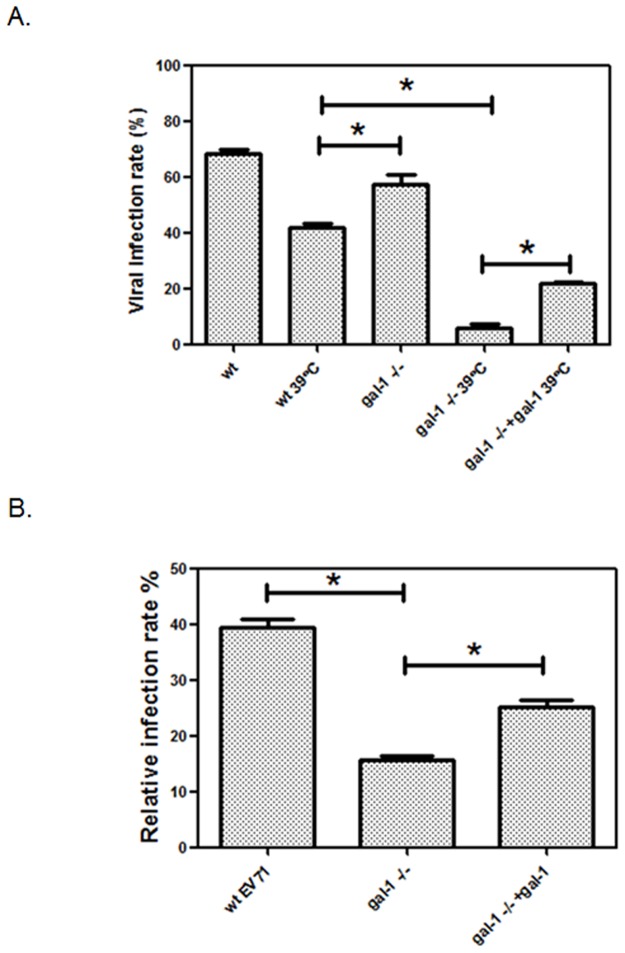
Galectin-1 facilitates EV71 viruses against thermal and environmental stress. WT EV71, galectin-1 -/- EV71 or galectin-1 -/- EV71 viruses with recombinant galectin-1 (25 ng/ml) were heated to 39°C for 4 h, and then infected SK-N-SH cells at 37°C (MOI = 0.5) for another 12 h. The infection rates of EV71 viruses were detected and analyzed by flow cytometry. (B) WT EV71, galectin-1 -/- EV71 or galectin-1 -/- EV71 viruses with recombinant galectin-1 (25 ng/ml) were stored at 4°C for 30 days, and then infected SK-N-SH cells at 37°C (MOI = 0.5) for 12 h. The relative infection rate was compared to freshly isolated EV71 virus of each group. *p<0.05 by one-way ANOVA followed by Tukey correction. Results are representative of two (B) or three (A) independent experiments.

In clinical practice, severe EV71-infected patients usually carry a high fever, as well as high virus titers, suggesting that EV71 viruses might be resistant to high temperatures.[3] Since EV71 viruses carried galectin-1 to support their infection, we next tried to investigate whether EV71 virus-bound galectin-1 was able to assist viruses against temperature-induced damage. Wild type or galectin-1-/- EV71 viruses were incubated at 39°C for 4 hours and then their infectivity to cells was evaluated. According the results shown in Fig. 6A, the infection rates for the wild type virus declined from 69% to 44% after heat treatment. However, infection rates of galectin-1-/- EV71 viruses dramatically dropped from 55.6% to 4.6% after heat treatment. Compared to wild type viruses, the viral particles of these galectin-1-/- EV71 viruses exhibited an intensified irregularity in their structures after heating them at 39°C (S2 Fig.). Furthermore, such a marked decline in infectivity of heat-treated galectin-1-/- EV71 viruses was recovered by a supplement of recombinant galectin-1. This indicates that EV71-bound galectin-1 can help viruses to resist temperature-induced damage. In addition to thermal stress, the role of galectin-1 in the EV71 virus long term stability was also examined. Wild type or galectin-1-/- EV71 viruses were stored at 4°C for 30 days and then their infectivity to cells was evaluated. Compared to viral infectivity since day 0, the infectivity of wild type EV71 viruses has declined by 60% on day 30. However, an almost 85% drop in infectivity occurred in galectin-1-/- EV71 viruses, which was partially reversed by the supplement of recombinant galectin-1 (Fig. 6B). These results indicate that EV71 viruses are resistant to thermal and environmental stress through the galectin-1 protein.

## Discussion

EV71 has a pentameric icosahedral outer capsid consisting of 60 copies of each of four structural proteins. These capsid proteins not only function as a receptor binding site, but also as the antigenic determinant recognized by the host antibody. We found that EV71 can bind to galectin-1 via VP1 and VP3. This binding occurs during viral replication within cells and also on released virions. EV71 virion-associated galectin-1 can facilitate the virus to efficiently attach and hence infect new host cells. Galectin-1-/- EV71 virus is shown to be less infectious and virulent in neuron cells and mice, respectively. It also is less stable in response to high temperature or long-term environmental stress without galectin-1, indicating the crucial role of galectin-1 in the EV71 replication cycle. Our findings suggest that the high level of serum galectin-1 in febrile patients might contribute to disease progress via maintaining EV71 virus infectivity and stability.

Fever is considered to be a host protective mechanism to limit virus replication because temperature is a key factor to mediate virus inactivation [21]. Thus, temperature sensitivity becomes a decisive factor for virus survival in hosts. Recent studies have also pointed out that the temperature sensitivity of EV71 virus may contribute to disease severity in EV71 infected patients. For instance, temperature resistant strains of EV71 are more infectious and virulent to primates than temperature sensitive ones[22]. Arita et al. reported that temperature sensitive mutant EV71 causes attenuated neuropathological changes and limited viral spread in monkeys[23]. More recently, Kung et al. revealed that EV71 viruses isolated from mild HFMD or herpangina patients are less resistant to high temperature (over 39°C) than those from severe neurological patients. They further demonstrated that amino acid sequence substitution of EV71 3D polymerase at 251 from isoleucine to threonine could result in reducing virus temperature sensitivity [24]. These studies suggested that EV71 viral genome mutation, particularly as in 3D polymerase, could help the virus to replicate within cells at a high temperature and hence promote neurological damage. However, how released EV71 viruses keep their stability to high temperature is poorly understood. Here we demonstrated that host cellular protein galectin-1 is associated with released EV71 virions to assist the virus against thermal and environmental stress. To our knowledge, this is the first report to reveal how EV71 virus utilizes a host factor to maintain its stability and infectivity.

Galectin constitutes a lectin family with a characteristic carbohydrate recognition domain of 130 amino acids. Mammalian galectins have been classified as proto, chimera or tandem repeat types. By recognizing carbohydrate moieties on the surface of viruses, bacteria, or fungi, galectin-1 functions as a pattern recognition receptor to defend against these pathogens, or in certain cases, it may serve as a stepping stone for their successful invasion. Hendra virus, Nipah virus, HIV virus and influenza virus have all been shown to interact with extracellular galectin-1. This interaction can interfere in Hendra or Nipah virus binding to host cells, but not in HIV or influenza virus[17,25]. Although the molecular mechanism that makes this difference is not clear, the glycan-dependent receptor binding between virus and host cells might be crucial. It is known that Nipah and Hendra virus bind their receptor by an extensive protein-protein interface, not via a glycan-dependent interaction as in HIV or influenza [17,25]. In this study, we also observed that galectin-1 interacts with EV71 via galactose to increase its binding on host cells. This indicates that glycan-dependent attachment between virus and host cells could be enhanced in the presence of galectin-1. EV71 is a non-enveloped virus which viral proteins are synthesized in the cytosol and theoretically poor glycosylated. However, in this study, we did find EV71 viruses have affinity to several plant lectins and galectin-1, suggesting that there are some glycans on the viruses. Since these viral capsids are less glycosylated, we speculated that other glycosylated molecules, such as glycoproteins, might mediate the interaction of lectins and EV71 viruses. Recent studies demonstrated that EV71 can bind to several host proteins, such as annexin II and HSP90, to enhance viral infectivity. Annexin II and HSP90 are known highly glycosylated proteins with N-acetyl glucosamine modification [26,27]. Our results suggested that N-acetyl glucosamine may mediate interaction between galectin-1 and EV71. Such glycoproteins with affinity to EV71 capsid proteins might be responsible for linking the lectins and viruses. That needs to further investigate.

Several human receptors have been reported as EV71 receptors. Using the cDNA library from the Jurkat T cell, which is susceptible to EV71 infection, to transfect into the resistant 293T cells, Nishimura *et al*., identified human P-selectin glycoprotein ligand-1 (PSGL-1) as a functional receptor[28]. With a similar approach of using genomic DNA from RD cells to transfect the L929 cells, Yamayoshi *et al*. identified another human scavenger receptor B2 (SCARB2) as a cellular receptor for EV71[29]. Furthermore, dendritic cell-specific intracellular adhesion molecules (DC-SIGN), heparin sulfate, annexin-II, HSP90 and vimentin have been shown to enhance EV71 infection [30–34].These studies indicate that EV71 may infect cells through interacting with different binding receptors and other accessory proteins. Here, we demonstrated that EV71 coupled with galectin-1 can increase both viral binding and infectivity. Our results suggest that the cell surface galectin-1 receptors might be responsible for this enhancement. Some of galectin-1 receptors, such as human brain galectin-1-binding polypeptide of 82 kDa (HBGp82) or GM-1 ganglioside, are highly expressed on neuron cells[35]. The contributing role of these galectin-1 receptors on EV71 infection is worth investigating further. The intracellular function of galectin-1 is different from classical surface or extracellular galectin-1 that interacts with glycan chains of glycoprotein in a carbohydrate-dependent manner. For the intracellular galectin-1, it can also bind oncogenic H-Ras to mediate Ras membrane anchorage and enhance the Ras activity [36,37]. In addition, galectin-1 or 3 can bind to ribonucleoprotein in the nucleus to participate in the splicing of pre-mRNA[38]. Recently Lin et al identified a nuclear ribonuclear protein K that binds the EV71 5′ untranslated region in virus replication[10]. Our findings that galectin-1 forms a complex with the EV71 virus suggest that intracellular galectin-1 might be involved in viral RNA synthesis.

Emerging evidence has implicated galectin-1 as a crucial regulator of immune cell function, especially for T cells. Activated T cells can secrete galactin-1 as an autocrine or paracrine regulator. Galectin-1 binding to glycosylated CD45, CD4, CD3, and CD2 on T cells as a counter receptor is reported to participate in T cell homeostasis, inhibit inflammation, modulate cytokine production, and induce apoptosis[39]. Accumulation of proapoptotic galectin-1 in immune privileged sites provides a protective mechanism from damage by infiltrating T cells[40]. In EV71 patients with pulmonary edema, peripheral T cells are dramatically dropped, suggesting that severe EV71 virus disease might be correlated with immunosuppression [41]. An *in vitro* study has suggested that EV 71 virus-induced Fas ligand expression might lead to a depletion of T cells in patients[42]. In this study, we showed that high a level of galectin-1 is presented in the serum of EV71 infected patients. Since galectin-1 shows high proapoptotic activity to T cells, it suggests that the high level galectin-1 in EV71 patients may contribute to T cell depletion and hence promote disease progression. According to our results, increased levels of serum galectin-1 were detected only in severe EV71-infected patients, not in non-severe EV71-infected patients, compared to control group. This suggests that serum level of galectin-1 may serve as a biomarker in severe EV71 infection. However, only limited numbers of patient specimens were tested in this study. We need to collect more clinical specimens to test this application of galectin-1 in EV71 infection. In conclusion, our findings uncover a new role of galectin-1 in facilitating EV71 virus infection and provide a potential therapeutic target for EV71 patients.

## Materials and Methods

### Cells, viruses, and reagents

RD (rhabdomyosarcoma) and SK-N-SH (human neuroblastoma) cells (ATCC) were maintained in Dulbecco’s modified Eagle’s medium (DMEM) containing 10% fetal bovine serum (FBS) with100 IU penicillin/ml and 100 mg streptomycin/ml. Enterovirus 71(EV71/Tainan/4643/98, GenBank accession number AF304458) was propagated in RD cells or SK-N-SH cells as described previously[43,44]. Recombinant human Gal-1 including a (His)^6^ tag sequence (rh-Gal-1) was expressed in *Escherichia coli* and purified to homogeneity[45]. The galectin-1 was dissolved in double-distilled water, and the working stock concentration was 1 mg/ml. The lectin activity of recombinant galectin-1 was also determined by monitoring the hemagglutination activity (see S3 Fig.). Plasmids pFLAG-MSCV-VP1, pFLAG-MSCV-VP2 and pFLAG-MSCV-VP3 were kindly provided by Dr. Shainn-Wei Wang of the Institute of Molecular Medicine, National Cheng Kung University.

### Patient Enrollment

Clinical specimens were collected from the Departments of Emergency Medicine and Pediatrics of National Cheng Kung University Hospital. Patients were confirmed virologically for EV71 infection. Total sera of 7 EV71 patients with aseptic meningitis, brain stem encephalitis or pulmonary edema were collected and grouped as severe EV71-infection. The sera of 5 EV71 patients with only HFMD or herpangina were collected and grouped as non-severe EV71-infection. Moreover, the sera of 12 healthy comparable volunteers were also collected to detect free form of galectin-1. This study was approved by the Clinical Research Ethics Committee of National Chen Kung University Hospital. Written informed consent was obtained from the participant’s parents or guardians.

### Generation of galectin-1-/- EV71 virus

Galectin-1 was silenced in SK-N-SH cells by stably expressing lentivirus-based shRNA, which targeted human galectin-1. The clone was obtained from the National RNAi Core Facility (Institute of Molecular Biology/Genomic Research Center, Academia Sinica, Taiwan). The target sequence for galectin-1 is TRCN000005742 5′-CCTGAATCTCAAACCTGGAGA-3′, and that for control luciferase is TRCN0000072247 5′-GAATCGTCGTATGCAGTGAAA-3′. The recombinant lentivirus was produced by co-transfection with two helper vectors, pCMVdeltaR8.91 and pMD.G, and a target vector pLKO.1-puro-shRNA to 293T cells. SK-N-SH cells were then infected with recombinant lentivirus for 24 hours, and stably expressed cells were selected by puromycin. The knockdown efficiency of target proteins was determined by Western blotting. To generate galecin-1-/- EV71 virus, the silenced SK-N-SH cells were infected with wild type EV71 virus at MOI = 5 for 12 h and then the released virus was collected. The virus title was further determined by plaque assay.

### ELISA and galectin-1 pull down assay

For galectin-1 binding, EV71 was added to a 96-well plate that was pre-coated with 0.4 μg/well of galectin-1, then the anti-EV71 antibody mAb979 (Millipore Corporation, USA) was added, followed by goat anti-mouse IgG antibody HRP-conjugate to detect the binding activity. The reaction was developed by TMB substrate (Clinical, Mansfield, MA) and the optical density at 450 nm (OD_450_) was determined. For galectin-1 to pull down EV71 virions, EV71(1×10^6^ PFU) was incubated with 5, 10, or 20 μg recombinant galectin-1 at 4°C overnight and TALON metal affinity resins were used to pull down galectin-1. The protein level of VP1 was detected by Western blotting with an anti-EV71 IgY (Genesis, Taiwan).

### Infection of EV71 in SK-N-SH cells or RD cells

SK-N-SH cells or RD cells were infected with EV71 at MOI = 1 for 8 h at 37°C and 5% CO_2_. The infected cells were fixed with 4% paraformaldehyde and permeabilized with 0.1% saponin on ice for 20 and 15 minutes, respectively. The viruses were detected by 1:1000 diluted anti-EV71 antibody (mAb979) and Alexa488 conjugated donkey anti-mouse IgG antibody and analyzed through flow cytometry. For detecting the cellular binding of EV71, SK-N-SH cells (5×10^4^ cells/tube) were incubated with wild type or galectin-1-/- EV71 at 4°C for 2 h. The unbound viruses were washed out and the viruses that bound to the cell surface were detected by anti-EV71 or anti-galectin-1 antibody, and then analyzed by flow cytometry.

### Plaque assay

SK-N-SH cells (2×10^5^ cells/well) were seeded in a 24-well plate to form a monolayer for 18 h, and the cells were then infected with serial diluted viruses. After 1 h incubation at 37°C, 1.6% methylcellulose with 2% FBS was added for further incubation at 37°C for 72 h. Crystal violet was overlaid to determine plaque formation.

### Immunofluorescent assay

For EV71 infection, SK-N-SH or RD cells (1×10^5^ cells) were incubated with EV71 at MOI = 1 for 8 h. The infected cells were fixed with methanol on ice for 10 minutes. The cells were stained with rabbit anti-galectin-1 antibody (Abcam Inc, USA) and anti-EV71 antibody (mAb3324, Millipore Corporation, USA) at 4°C for 18 h. After washing, Alexa488-conjugated goat anti-rabbit IgG antibody and TRITC conjugated goat anti-mouse IgG antibody were used to detect galectin-1 and EV71, respectively. The cell nuclei were stained with Hoechst 33258. The co-localization of galectin-1 and EV71 was analyzed using confocal microscopy.

### Western blotting and immunoprecipitation assay

Total proteins of EV71-infeced SK-N-SH or RD cells were extracted with lysis buffer (20 mM Tris-HCl pH 7.5, 150 mM NaCl, 1 mM Na2EDTA, 1 mM EGTA, 1% Triton, 2.5 mM sodium pyrophosphate, 1 mM beta-glycerophosphate, 1 mM Na3VO4, 1 μg/ml leupeptin). Proteins were separated by SDS-PAGE and transferred to PVDF membranes. The membranes were blocked and incubated with primary antibodies. After incubation with peroxidase-conjugated secondary antibodies, the blots were visualized by enhancing chemiluminescence reagents (PerkinElimer Life Sciences, Boston, MA). For the galectin-1 or EV71 immunoprecipitation assay, cells were extracted by lysis buffer and the extracted proteins were then collected. Anti-galectin-1 or EV71 antibody and protein G-Sepharose beads were added to the extracted proteins at 4°C. The beads were isolated and washed by centrifugation and the bound proteins were determined by Western blotting as described above.

### EV71 infection in mice

C56BL/6 mice were raised and cared for according to the guidelines set up by the National Science Council, ROC. The mouse experiments were approved by the institutional animal care and use committee of National Cheng Kung University (Permit Number: 100068). One-week-old C56BL/6 mice were infected with 10^6^ PFU of wild type or galectin-1-/- EV71 by intraperitoneal injection. Mice were observed twice daily for clinical illness and death as previous described [46]. Clinical illness was graded as follows: 0, healthy; 1, ruffled fur and hunchbacked appearance; 2, wasting; 3, limb weakness; 4, limb paralysis; and 5, moribund and death. The mortality was monitored every day post infection. The survival rate was found to be significantly different at p< 0.01 according to a Log-rank (Mantel-Cox) Test.

### Statistics

Statistical analysis was performed by using Graphpad Prism software. Differences in groups of patient’s serum galectin-1 were compared using Kruskal-Wallis one-way analysis of variance (ANOVA) with Dunn’s posttest. Log-rank (Mantel-Cox) Test was used to analyze differences in group survival rates. Other experimental data were analyzed using one-way analysis of variance (ANOVA) followed by Tukey’s multiple-comparison posttest. A P value less than 0.05 was considered significant.

## Supporting Information

S1 Fig EV71 virus binds to various lectins. EV71 viruses (1×10^6^ PFU) were added to ELISA plate coated with different lectins such as Con A, LCA, RCA, DBA or WGA. The bound virus was detected by anti-EV71 antibody and HRP-conjugated goat anti-mouse IgG antibody.
(TIF)

S2 Fig Electronic micrographs of WT and gal-1-/- EV71 viruses. WT or gal-1-/- EV71 viruses were either heated at 39°C for 1 hour or not. The structures of these viruses were observed by electron microscopy (×60,000). Arrows indicate contact viruses; arrow heads indicate disrupted viruses.
(TIF)

S3 Fig Hemagglutination of human blood cells by recombinant galectin-1. Hemagglutination of human O-type blood cells by lectins was performed in a 96-well microtiter plate. Serious dilution of plant lectin Con A and recombinant galectin-1 were added to the 96-well plate containing 0.05% blood cells for 2 hours at room temperature. The agglutination activity was determined on the sedimentary state of the blood cells.
(TIF)

## References

[1] Schmidt NJ, Lennette EH, Ho HH (1974) An apparently new enterovirus isolated from patients with disease of the central nervous system. J Infect Dis 129: 304–309. 4361245 10.1093/infdis/129.3.304

[2] Ho M, Chen ER, Hsu KH, Twu SJ, Chen KT, et al (1999) An epidemic of enterovirus 71 infection in Taiwan. Taiwan Enterovirus Epidemic Working Group. N Engl J Med 341: 929–935. 10498487 10.1056/NEJM199909233411301

[3] Wang SM, Liu CC, Tseng HW, Wang JR, Huang CC, et al (1999) Clinical spectrum of enterovirus 71 infection in children in southern Taiwan, with an emphasis on neurological complications. Clin Infect Dis 29: 184–190. 10433583 10.1086/520149

[4] Liu CC, Tseng HW, Wang SM, Wang JR, Su IJ (2000) An outbreak of enterovirus 71 infection in Taiwan, 1998: epidemiologic and clinical manifestations. J Clin Virol 17: 23–30. 10814935 10.1016/s1386-6532(00)00068-8

[5] Wong SS, Yip CC, Lau SK, Yuen KY (2010) Human enterovirus 71 and hand, foot and mouth disease. Epidemiol Infect 138: 1071–1089. 10.1017/S0950268809991555 20056019

[6] Brown BA, Pallansch MA (1995) Complete nucleotide sequence of enterovirus 71 is distinct from poliovirus. Virus Res 39: 195–205. 8837884 10.1016/0168-1702(95)00087-9

[7] Lin JY, Shih SR, Pan M, Li C, Lue CF, et al (2009) hnRNP A1 interacts with the 5′ untranslated regions of enterovirus 71 and Sindbis virus RNA and is required for viral replication. J Virol 83: 6106–6114. 10.1128/JVI.02476-08 19339352 PMC2687368

[8] Lin JY, Li ML, Shih SR (2009) Far upstream element binding protein 2 interacts with enterovirus 71 internal ribosomal entry site and negatively regulates viral translation. Nucleic Acids Res 37: 47–59. 10.1093/nar/gkn901 19010963 PMC2615614

[9] Huang PN, Lin JY, Locker N, Kung YA, Hung CT, et al (2011) Far upstream element binding protein 1 binds the internal ribosomal entry site of enterovirus 71 and enhances viral translation and viral growth. Nucleic Acids Res 39: 9633–9648. 10.1093/nar/gkr682 21880596 PMC3239202

[10] Lin JY, Li ML, Huang PN, Chien KY, Horng JT, et al (2008) Heterogeneous nuclear ribonuclear protein K interacts with the enterovirus 71 5′ untranslated region and participates in virus replication. J Gen Virol 89: 2540–2549. 10.1099/vir.0.2008/003673-0 18796723

[11] Tang WF, Yang SY, Wu BW, Jheng JR, Chen YL, et al (2007) Reticulon 3 binds the 2C protein of enterovirus 71 and is required for viral replication. J Biol Chem 282: 5888–5898. 17182608 10.1074/jbc.M611145200

[12] Wang RY, Kuo RL, Ma WC, Huang HI, Yu JS, et al (2013) Heat shock protein-90-beta facilitates enterovirus 71 viral particles assembly. Virology 443: 236–247. 10.1016/j.virol.2013.05.001 23711381

[13] Weng KF, Li ML, Hung CT, Shih SR (2009) Enterovirus 71 3C protease cleaves a novel target CstF-64 and inhibits cellular polyadenylation. PLoS Pathog 5: e1000593 10.1371/journal.ppat.1000593 19779565 PMC2742901

[14] Leffler H, Carlsson S, Hedlund M, Qian Y, Poirier F (2004) Introduction to galectins. Glycoconj J 19: 433–440. 14758066 10.1023/B:GLYC.0000014072.34840.04

[15] Camby I, Le Mercier M, Lefranc F, Kiss R (2006) Galectin-1: a small protein with major functions. Glycobiology 16: 137R–157R. 16840800 10.1093/glycob/cwl025

[16] St-Pierre C, Ouellet M, Tremblay MJ, Sato S (2010) Galectin-1 and HIV-1 Infection. Methods Enzymol 480: 267–294. 10.1016/S0076-6879(10)80013-8 20816214

[17] St-Pierre C, Manya H, Ouellet M, Clark GF, Endo T, et al (2011) Host-soluble galectin-1 promotes HIV-1 replication through a direct interaction with glycans of viral gp120 and host CD4. J Virol 85: 11742–11751. 10.1128/JVI.05351-11 21880749 PMC3209312

[18] Garner OB, Aguilar HC, Fulcher JA, Levroney EL, Harrison R, et al (2010) Endothelial galectin-1 binds to specific glycans on nipah virus fusion protein and inhibits maturation, mobility, and function to block syncytia formation. PLoS Pathog 6: e1000993 10.1371/journal.ppat.1000993 20657665 PMC2904771

[19] Yang ML, Chen YH, Wang SW, Huang YJ, Leu CH, et al (2011) Galectin-1 binds to influenza virus and ameliorates influenza virus pathogenesis. J Virol 85: 10010–10020. 10.1128/JVI.00301-11 21795357 PMC3196456

[20] Su PY, Liu YT, Chang HY, Huang SW, Wang YF, et al (2012) Cell surface sialylation affects binding of enterovirus 71 to rhabdomyosarcoma and neuroblastoma cells. BMC Microbiol 12: 162 10.1186/1471-2180-12-162 22853823 PMC3478995

[21] Bertrand I, Schijven JF, Sanchez G, Wyn-Jones P, Ottoson J, et al (2012) The impact of temperature on the inactivation of enteric viruses in food and water: a review. J Appl Microbiol 112: 1059–1074. 10.1111/j.1365-2672.2012.05267.x 22380614

[22] Hashimoto I, Hagiwara A (1983) Comparative studies on the neurovirulence of temperature-sensitive and temperature-resistant viruses of enterovirus 71 in monkeys. Acta Neuropathol 60: 266–270. 6310928 10.1007/BF00691875

[23] Arita M, Shimizu H, Nagata N, Ami Y, Suzaki Y, et al (2005) Temperature-sensitive mutants of enterovirus 71 show attenuation in cynomolgus monkeys. J Gen Virol 86: 1391–1401. 15831951 10.1099/vir.0.80784-0

[24] Kung YH, Huang SW, Kuo PH, Kiang D, Ho MS, et al (2010) Introduction of a strong temperature-sensitive phenotype into enterovirus 71 by altering an amino acid of virus 3D polymerase. Virology 396: 1–9. 10.1016/j.virol.2009.10.017 19906393

[25] Levroney EL, Aguilar HC, Fulcher JA, Kohatsu L, Pace KE, et al (2005) Novel innate immune functions for galectin-1: galectin-1 inhibits cell fusion by Nipah virus envelope glycoproteins and augments dendritic cell secretion of proinflammatory cytokines. J Immunol 175: 413–420. 15972675 10.4049/jimmunol.175.1.413 PMC4428613

[26] Phueaouan T, Chaiyawat P, Netsirisawan P, Chokchaichamnankit D, Punyarit P, et al (2013) Aberrant O-GlcNAc-modified proteins expressed in primary colorectal cancer. Oncol Rep 30: 2929–2936. 10.3892/or.2013.2794 24126823

[27] Overath T, Kuckelkorn U, Henklein P, Strehl B, Bonar D, et al (2012) Mapping of O-GlcNAc sites of 20 S proteasome subunits and Hsp90 by a novel biotin-cystamine tag. Mol Cell Proteomics 11: 467–477. 10.1074/mcp.M111.015966 22556278 PMC3412975

[28] Nishimura Y, Shimojima M, Tano Y, Miyamura T, Wakita T, et al (2009) Human P-selectin glycoprotein ligand-1 is a functional receptor for enterovirus 71. Nat Med 15: 794–797. 10.1038/nm.1961 19543284

[29] Yamayoshi S, Yamashita Y, Li J, Hanagata N, Minowa T, et al (2009) Scavenger receptor B2 is a cellular receptor for enterovirus 71. Nat Med 15: 798–801. 10.1038/nm.1992 19543282

[30] Lin YW, Wang SW, Tung YY, Chen SH (2009) Enterovirus 71 infection of human dendritic cells. Exp Biol Med (Maywood) 234: 1166–1173. 10.3181/0903-RM-116 19596831

[31] Tan CW, Poh CL, Sam IC, Chan YF (2013) Enterovirus 71 uses cell surface heparan sulfate glycosaminoglycan as an attachment receptor. J Virol 87: 611–620. 10.1128/JVI.02226-12 23097443 PMC3536405

[32] Yang SL, Chou YT, Wu CN, Ho MS (2011) Annexin II binds to capsid protein VP1 of enterovirus 71 and enhances viral infectivity. J Virol 85: 11809–11820. 10.1128/JVI.00297-11 21900167 PMC3209289

[33] Du N, Cong H, Tian H, Zhang H, Zhang W, et al (2014) Cell Surface Vimentin is an Attachment Receptor for Enterovirus 71. J Virol. 10.1128/JVI.03826-13 PMC4019121 24623428

[34] Tsou YL, Lin YW, Chang HW, Lin HY, Shao HY, et al (2013) Heat shock protein 90: role in enterovirus 71 entry and assembly and potential target for therapy. PLoS One 8: e77133 10.1371/journal.pone.0077133 24098578 PMC3788750

[35] Elola MT, Chiesa ME, Alberti AF, Mordoh J, Fink NE (2005) Galectin-1 receptors in different cell types. J Biomed Sci 12: 13–29. 15864736 10.1007/s11373-004-8169-5

[36] Elad-Sfadia G, Haklai R, Ballan E, Gabius HJ, Kloog Y (2002) Galectin-1 augments Ras activation and diverts Ras signals to Raf-1 at the expense of phosphoinositide 3-kinase. J Biol Chem 277: 37169–37175. 12149263 10.1074/jbc.M205698200

[37] Paz A, Haklai R, Elad-Sfadia G, Ballan E, Kloog Y (2001) Galectin-1 binds oncogenic H-Ras to mediate Ras membrane anchorage and cell transformation. Oncogene 20: 7486–7493. 11709720 10.1038/sj.onc.1204950

[38] Vyakarnam A, Dagher SF, Wang JL, Patterson RJ (1997) Evidence for a role for galectin-1 in pre-mRNA splicing. Mol Cell Biol 17: 4730–4737. 9234729 10.1128/mcb.17.8.4730 PMC232325

[39] Liu SD, Tomassian T, Bruhn KW, Miller JF, Poirier F, et al (2009) Galectin-1 tunes TCR binding and signal transduction to regulate CD8 burst size. J Immunol 182: 5283–5295. 10.4049/jimmunol.0803811 19380775

[40] Rabinovich GA (1999) Galectins: an evolutionarily conserved family of animal lectins with multifunctional properties; a trip from the gene to clinical therapy. Cell Death Differ 6: 711–721. 10467344 10.1038/sj.cdd.4400535

[41] Wang SM, Lei HY, Huang KJ, Wu JM, Wang JR, et al (2003) Pathogenesis of enterovirus 71 brainstem encephalitis in pediatric patients: roles of cytokines and cellular immune activation in patients with pulmonary edema. J Infect Dis 188: 564–570. 12898444 10.1086/376998

[42] Chen LC, Shyu HW, Chen SH, Lei HY, Yu CK, et al (2006) Enterovirus 71 infection induces Fas ligand expression and apoptosis of Jurkat cells. J Med Virol 78: 780–786. 16628611 10.1002/jmv.20623

[43] Chen CS, Yao YC, Lin SC, Lee YP, Wang YF, et al (2007) Retrograde axonal transport: a major transmission route of enterovirus 71 in mice. J Virol 81: 8996–9003. 17567704 10.1128/JVI.00236-07 PMC1951457

[44] Chen YC, Yu CK, Wang YF, Liu CC, Su IJ, et al (2004) A murine oral enterovirus 71 infection model with central nervous system involvement. J Gen Virol 85: 69–77. 14718621 10.1099/vir.0.19423-0

[45] Hsieh SH, Ying NW, Wu MH, Chiang WF, Hsu CL, et al (2008) Galectin-1, a novel ligand of neuropilin-1, activates VEGFR-2 signaling and modulates the migration of vascular endothelial cells. Oncogene 27: 3746–3753. 10.1038/sj.onc.1211029 18223683

[46] Liu ML, Lee YP, Wang YF, Lei HY, Liu CC, et al (2005) Type I interferons protect mice against enterovirus 71 infection. J Gen Virol 86: 3263–3269. 16298971 10.1099/vir.0.81195-0

