# Supplementary figures and images for: Enterovirus 71 Virion-Associated Galectin-1 Facilitates Viral Replication and Stability
Source: PLoS One. 2015 Feb 23;10(2):e0116278. doi: 10.1371/journal.pone.0116278 (PMC4338065; doi:10.1371/journal.pone.0116278)

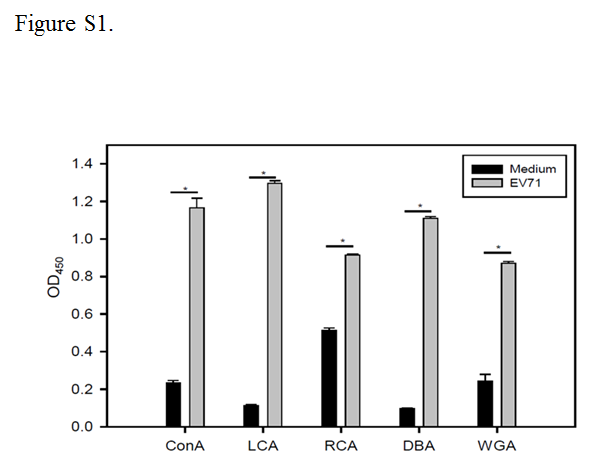

Supplement: S1 Fig — EV71 viruses (1×10^6^ PFU) were added to ELISA plate coated with different lectins such as Con A, LCA, RCA, DBA or WGA. The bound virus was detected by anti-EV71 antibody and HRP-conjugated goat anti-mouse IgG antibody. (TIF) [file pone.0116278.s001.TIF]

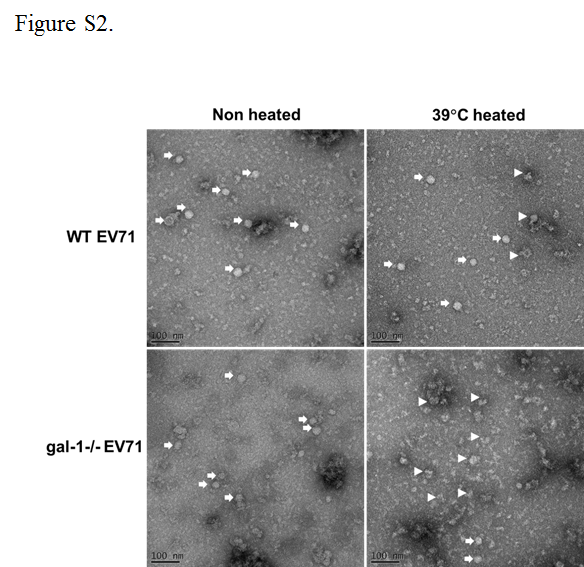

Supplement: S2 Fig — WT or gal-1-/- EV71 viruses were either heated at 39°C for 1 hour or not. The structures of these viruses were observed by electron microscopy (×60,000). Arrows indicate contact viruses; arrow heads indicate disrupted viruses. (TIF) [file pone.0116278.s002.TIF]

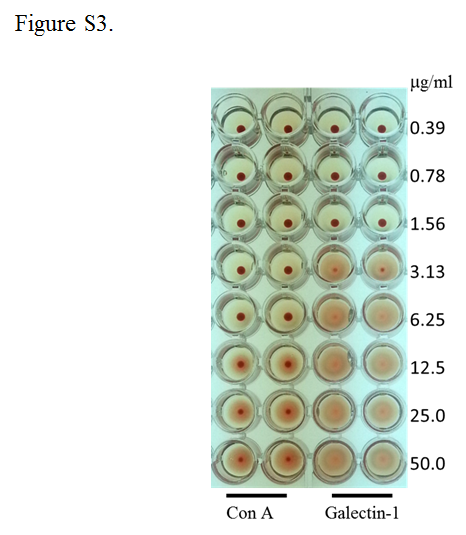

Supplement: S3 Fig — Hemagglutination of human O-type blood cells by lectins was performed in a 96-well microtiter plate. Serious dilution of plant lectin Con A and recombinant galectin-1 were added to the 96-well plate containing 0.05% blood cells for 2 hours at room temperature. The agglutination activity was determined on the sedimentary state of the blood cells. (TIF) [file pone.0116278.s003.TIF]
